# Overcoming Resistance of Human Non-Hodgkin’s Lymphoma to CD19-CAR CTL Therapy by Celecoxib and Histone Deacetylase Inhibitors

**DOI:** 10.3390/cancers10060200

**Published:** 2018-06-14

**Authors:** Antoni Xavier Torres-Collado, Ali R. Jazirehi

**Affiliations:** Department of Surgery, Division of Surgical Oncology, Jonsson Comprehensive Cancer Center, David Geffen School of Medicine, University of California, Los Angeles (UCLA), Los Angeles, CA 90095, USA; axtorres@gmail.com

**Keywords:** chimeric antigen receptor, non-Hodgkin’s lymphoma, adoptive cell transfer, apoptosis, signal transduction, immunotherapy, resistance, B cell hematological malignancies, Celebrex, histone deacetylase inhibitor

## Abstract

Patients with B-cell non-Hodgkin’s lymphoma (B-NHL) who fail to respond to first-line treatment regimens or develop resistance, exhibit poor prognosis. This signifies the need to develop alternative treatment strategies. CD19-chimeric antigen receptor (CAR) T cell-redirected immunotherapy is an attractive and novel option, which has shown encouraging outcomes in phase I clinical trials of relapsed/refractory NHL. However, the underlying mechanisms of, and approaches to overcome, acquired anti-CD19CAR CD8^+^ T cells (CTL)-resistance in NHL remain elusive. CD19CAR transduced primary human CTLs kill CD19^+^ human NHLs in a CD19- and caspase-dependent manner, mainly via the tumor necrosis factor-related apoptosis-inducing ligand (TRAIL) apoptotic pathway. To understand the dynamics of the development of resistance, we analyzed several anti-CD19CAR CTL-resistant NHL sublines (R-NHL) derived by serial exposure of sensitive parental lines to excessive numbers of anti-CD19CAR CTLs followed by a limiting dilution analysis. The R-NHLs retained surface CD19 expression and were efficiently recognized by CD19CAR CTLs. However, R-NHLs developed cross-resistance to CD19CAR transduced human primary CTLs and the Jurkat human T cell line, activated Jurkat, and lymphokine activated killer (LAK) cells, suggesting the acquisition of resistance is independent of CD19-loss and might be due to aberrant apoptotic machinery. We hypothesize that the R-NHL refractoriness to CD19CAR CTL killing could be partially rescued by small molecule sensitizers with apoptotic-gene regulatory effects. Chromatin modifiers and Celecoxib partially reversed the resistance of R-NHL cells to the cytotoxic effects of anti-CD19CAR CTLs and rhTRAIL. These in vitro results, though they require further examination, may provide a rational biological basis for combination treatment in the management of CD19CAR CTL-based therapy of NHL.

## 1. Introduction

The standard treatment option for non-Hodgkin’s lymphoma (NHL) patients includes the chemotherapy regimen CHOP (cyclophosphamide, doxorubicin, vincristine, and prednisone), which only induces a complete response (CR) in 40% of elderly patients, has an overall survival rate of only 35% [1], and is very toxic [2]. The most effective strategy to increase CHOP efficacy is to combine it with rituximab [R-CHOP] [3]. Rituximab is an effective single-agent in indolent lymphoma patients [4]. A 76% CR rate has been reported in patients treated with R-CHOP compared to a 63% CR rate in patients treated with CHOP alone [5]. R-CHOP treatment has also shown promise in younger patients. Two groups of 18 to 60-year-olds were given either CHOP or R-CHOP; the R-CHOP group had a 79% 3-year event-free survival rate compared to a 59% 3-year event-free survival rate in the CHOP-only group [6]. Although R-CHOP is superior to CHOP treatment, NHLs can eventually develop resistance to this regimen. Chimeric Antigen Receptor (CAR)-engineered T cells targeted at various tumor antigens were developed to circumvent acquired resistance. This method of immunotherapy reduced the severity of adverse effects (AE) and increased the specificity for tumor-associated antigens.

CAR T cells recognize tumors in an MHC-independent style, and thus are advantageous over TCR-engineered T cell Adoptive Cell Transfer (ACT) [7]. A CAR typically contains a ligand-binding domain, such as a single-chain variable fragment (scFv) derived from an mAb or an Ag-binding fragment (Fab) and the signaling domain CD3ζ which serves to trigger T cell activation [8,9]. Three generations of CAR T cells have been developed. The first generation consists of only a ligand-binding domain and a signaling domain [10]. The second and third generations use various co-stimulatory domains to enhance T cells’ specificity, proliferation, and cytokine production [11]. In second generation CAR T cells, the activation domain is fused with a co-stimulatory domain, such as CD28, 4-1BB, OX40 or DAP10 [8]. Dual-signaling CAR T cells enhance the strength of signaling as well as in vivo persistence [12]. Third generation CAR T cells have a second costimulatory domain added to the primary costimulatory domain (e.g., CD28/4-1BB/CD3ζ), which enhances the cytotoxic potential and effector functions of T cells, including proliferation, expansion, and cytokine production [13,14]. 

The most successful clinical applications of CAR therapy have been in the CD19-targeted treatment of B cell malignancies [15]. CD19 is an ideal target, as it is expressed in B-cell leukemia, lymphomas, and normal B cells, but is absent in other cell types [16,17]. All trials have encompassed a conditioning chemotherapy regimen prior to infusion, which greatly augments CAR T cell persistence and antitumor potential [18]. The antitumor activity of anti-CD19 CAR T cells was first reported in advanced follicular NHL, which resulted in dramatic regression. Peripheral blood B-cells were absent for approximately 39 weeks post-infusion, yet no acute toxicities arose [19]. A similar trial was conducted on eight patients with advanced, progressive B cell malignancies, six of whom obtained remissions. Four patients experienced extended B cell depletion, and four of the eight had elevated IFN-γ and TNF, with correlating acute toxicities [20]. Numerous additional clinical trials have resulted in partial or complete remissions in CLL, B cell ALL, and NHL receiving autologous CD19-redirected CAR T cells. Furthermore, anti-CD19 CAR T cell therapy has shown no persistent toxicities except for transient B-cell aplasia [20,21,22,23,24,25,26,27]. 

In eukaryotic cells, histone acetylation and deacetylation are processes that are central to transcription regulation [28]. The balance between these two processes is required for normal cell growth, and histone acetyltransferases and deacetylases are involved in the development of several diseases, including cancer [29]. Histone deacetylase inhibitors (HDACis) specifically target histone deacetylases and alter gene transcription selectively [30]. Hydroxamic acid-based vorinostat, also known as suberoylanilide hydroxamic acid (SAHA) or Zolinza, inhibits class I, II, and IV deacetylases [28]. The ability of HDACis to regulate apoptotic genes makes them effective anti-tumor agents. Tumor necrosis factor-related apoptosis-inducing ligand (TRAIL) induces apoptosis in tumor cells, yet, leaves untransformed cells mostly unaffected [31]. HDACis have been shown to upregulate death receptor 5 (DR5), a receptor of TRAIL, and the combined use of HDACis and TRAIL was demonstrated to be capable of inducing cleavage of Bid and caspases-3, -8, -9, -10, all of which are involved in extrinsic apoptotic pathways [32]. HDACis also induce apoptosis via intrinsic pathways. They reduce the expression of key antiapoptotic proteins, such as Bcl-_xL_, Mcl-1, and XIAP, and increase the expression of the proapoptotic proteins Bim, Bax, and Bak [33]. 

Another potent HDACi that is capable of inhibiting class I, II, and IV HDACis is panobinostat, also known as LBH589 [34]. LBH589 interferes with several processes involved in cell cycle regulation and apoptosis, thus hindering tumor growth. By downregulating phosphorylated signal transducer and activator of transcription (STAT) 3 and STAT 5, LBH589 can also induce the cytotoxicity of SAHA-resistant cutaneous T-cell lymphoma (CTCL) [35]. 

Celecoxib, also known as Celebrex, is a nonsteroidal anti-inflammatory drug (NSAID), commonly used to treat rheumatic diseases. It is also being used to treat several types of malignancies, including breast, colorectal, and prostate cancers, due to its ability to inhibit cyclooxygenase-2 (Cox-2) [36,37,38]. Cox-2 facilitates tumor growth via its enzymatic product, prostaglandin E2 (PGE_2_) [39]. PGE_2_ interacts with several other proteins to create an environment that is favorable for tumor development. It induces the expression of the angiogenesis regulators interleukin-6 (IL-6) and haptoglobin and upregulates the expression of Mcl-1 via PI3K/Akt-dependent pathways [40,41]. By inhibiting Cox-2, celecoxib can indirectly inhibit the expression of PGE_2_, thus reducing tumor growth. Furthermore, celecoxib downregulates Mcl-1 expression, allowing the proapoptotic proteins Bax and Bak to initiate the intrinsic apoptotic pathway by triggering the release of cytochrome c [42,43]. In Cox-2-deficient gastric cancer cells, celecoxib induces caspase-dependent apoptosis via the Akt/GSK3β/NAG-1 pathway; it inhibits Akt phosphorylation, and GSK3β, a downstream target of Akt, and upregulates NAG-1 expression, a pro-apoptotic and anti-tumorigenic protein [44]. Celecoxib also downregulates the expression of survivin, a multifunctional IAP family member that interferes with caspase activation [45]. 

Although anti-CD19CAR T cell-based therapy has shown encouraging results in NHL clinical trials, the underlying mechanism of resistance acquisition following initial treatment and approaches to overcome resistance remain elusive. The goals of this study were to establish an in vitro model of resistance of human NHL cells to CD19CAR transduced primary human CTLs, to gain a deeper understanding of potential resistance mechanisms and to design approaches to reverse resistance. Our results suggest that CD19CAR transduced primary human CTLs kill CD19^+^ human NHLs in a CD19- and caspase-dependent manner, mainly via the TRAIL apoptotic pathway. Serial exposure of sensitive parental lines to excessive numbers of anti-CD19CAR CTLs for 8 weeks, followed by a limiting dilution analysis yielded several anti-CD19CAR CTL-resistant NHL sublines (R-NHL), which retained surface CD19 expression, and were efficiently recognized by CD19CAR CTLs. However, R-NHLS developed cross-resistance not only to CD19CAR transduced human primary CTLs, but also to various other immune effector cells. These results suggest that the acquisition of resistance is independent of downregulation/loss of CD19 and is presumably due to deregulated apoptotic machinery. Our results further suggest that the unresponsiveness of R-NHL to anti-CD19 CAR CTL- and TRAIL-mediated killing is amenable to various small molecule sensitizing agents which have regulatory effects on apoptotic gene products; resistance could partially be rescued by SAHA, LBH589, and celecoxib. The in vitro results presented in this study, while they require further examination, may provide a rational biological/molecular basis for the incorporation of immune sensitizers into CD19CAR CTL-based protocols of NHL patients. 

## 2. Results

### 2.1. CD19CAR-Transduced Primary Human CTLs Kill CD19^+^ Human Non-Hodgkin’s Lymphoma (NHL) Cell Lines

**Figure 1:**
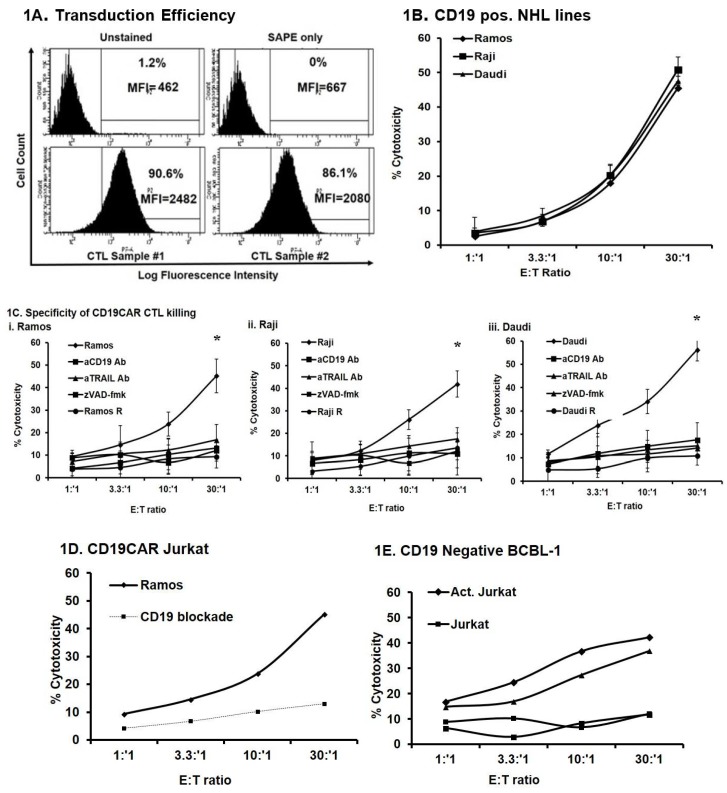
CD19 chimeric antigen receptor (CAR) transduced primary human CD8^+^ T cells (CTLs) kill CD19^+^ human non-Hodgkin’s lymphoma (NHL) cell lines. (**A**) The transduction efficiency of CD19CAR transduced lymphocytes was measured by fluorescence-activated cell sorting (FACS) analysis as detailed in the Materials and Methods section. The results are representative of at least two independent experiments); (**B**) The sensitivity of various CD19^+^ NHL lines (Ramos, Raji, Daudi) to CD19CAR CTL killing; (**C**) CD19CAR CTLs kill NHL cells in a CD19-specific, and caspase- and tumor necrosis factor-related apoptosis-inducing ligand (TRAIL)-mediated manner. Ramos NHL cells were left either untreated or pretreated with TRAIL, CD19 blocking mAb or zVAD-fmk (1.0 μg/mL, 6 h) and were used in a ^51^Cr-release assay using CD19CAR CTLs as effectors. BCBL-1 NHL cells were used as a negative control; (**D**) The specificity of CD19CAR Jurkat killing of NHL. The killing of NHL cells by CD19CAR transduced Jurkat cells (sorted to 100% purity) was significantly reduced by pretreatment of the cells with anti-CD19 mAb (1 μg/mL, 6 h). (**E**) Killing of the CD19 negative NHL B-cell line, BCBL-1, by various immune effector cells. * *p* values < 0.05 are considered to be significant.

Human peripheral blood CD8^+^ T cells (CTL), transduced to high efficiency (>90%) with a retroviral vector encoding CD19CAR (Figure 1A), efficiently recognized and killed three representative CD19^+^ NHL cell lines, Ramos, Raji, and Daudi (Figure 1B). NHLs were predominantly killed in a caspase- and CD19-dependent manner through the TRAIL apoptotic pathway as blocking antibodies to caspases, CD19, and TRAIL inhibited killing. CD19CAR CTLs failed to kill the CD19^−^ BCBL-1 line. (Figure 1C). We subsequently transduced the Jurkat human T cell line with the CD19CAR construct, followed by cell sorting to 100% purity. CD19CAR Jurkat T cells killed Ramos which was reduced by the CD19 blockade (Figure 1D). High dose IL-2 [(3000 IU/mL)/αCD3 (50 ng/mL)]-activated, non-transduced Jurkat or activated, CD19CAR transduced Jurkat cells killed BCBL-1 cells. CD19CAR CTLs or non-activated Jurkat cells failed to kill BCBL-1 cells (Figure 1E). These results show that CD19CAR transduced primary human CTLs and the Jurkat T cell line efficiently kill NHL lines.

### 2.2. Generation of CD19CAR CTL Resistant (R)-NHL Sublines

**Figure 2:**
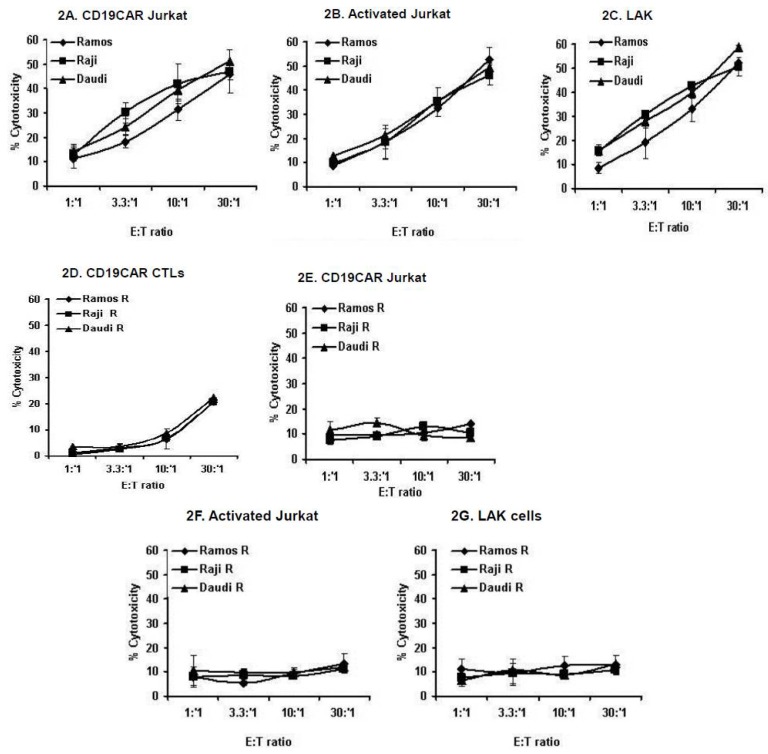
Generation of CD19CAR CTL-resistant (R)-NHL sublines. The sensitivity of NHL lines to killing mediated by various immune effector cells. (**A**) CD19CAR Jurkat (sorted to 100% purity); (**B**) activated (non-transduced Jurkat T cell line); (**C**) lymphokine activated killer (LAK) cells. Cross-resistance of CD19CAR CTL-resistant NHL to various immune effector cells; (**D**) CD19CAR CTL; (**E**) CD19CAR Jurkat (sorted to 100% purity); (**F**) activated (non-transduced) Jurkat T cell line (3000 IU/mL IL-2); (**G**) lymphokine activated killer (LAK) cells. NHL cells (Ramos, Raji, Daudi) were labeled with ^51^Chromium (1 h, 37 °C), washed 2× with ice-cold PBS and used in standard ^51^Cr-release assay. *p* values < 0.05 are considered to be significant.

We tested the ability of various effector cells to kill NHL lines. Three subsets of immune cells were used in standard cytotoxicity assays as effectors: CD19CAR-Jurkat cells sorted to 100% purity (which kill through CD19 recognition), activated (3000 IU/mL IL-2 + 50 ng/mL αCD3 mAb) Jurkat and lymphokine activated killer (LAK) cells (both of which kill indiscriminately and independently of CD19 or MHC) efficiently killed NHL lines, albeit to varying degrees (Figure 2A–C).

The serial exposure of NHL lines to increasing numbers of CD19CAR CTLs over a two-month period followed by a limiting dilution analysis to obtain a homogenous population yielded multiple NHL sublines that were resistant to the cytostatic effects of CD19CAR transduced primary human CTLs and the Jurkat T cell line (Figure 2D,E). These results show that CD19CAR CTL-resistant NHLs develop cross-resistance to the cytotoxic effects of CD19CAR-Jurkat cells, suggesting the use of a common apoptotic pathway by CD19CAR transduced primary human CTLs and the T cell line in killing NHL cells.

Next, we tested the ability of additional immune effector cells to kill CD19CAR CTL-resistant NHL cells. The CD19CAR CTL-resistant NHLs (also resistant to CD19CAR Jurkat) used as targets were co-cultured with activated Jurkat and LAK cells as effectors in standard cytotoxicity assays. CD19CAR CTL-resistant NHLs exhibited resistance to the cytotoxic effects of activated Jurkat and LAK cells (Figure 2F,G). These results suggest the existence of a shared apoptotic pathway used by CD19CAR CTLs, activated Jurkat, and LAK immune effector cells in killing NHL cells.

### 2.3. Recognition of Resistant-NHL Sublines by CD19CAR CTLs

**Figure 3:**
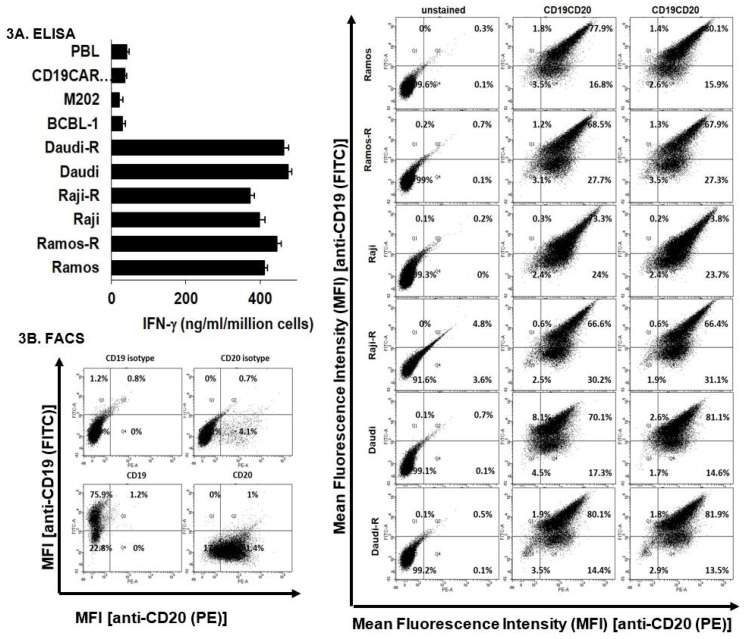
Recognition of resistant-NHL sublines by CD19CAR CTLs. (**A**) Surface expression of CD19 and CD20 on NHL lines: NHL cells (10^6^) were either stained with CD19 and CD20 isotype control or fluorochrome labeled anti-CD19 [FITC]/anti-CD20 [PE] mAbs, as detailed in the Materials and Methods section and subjected to FACS analysis. The results of two independent experiments are presented; (**B**) Recognition of NHL cells by CD19CAR CTL: 10^6^ tumors were co-incubated overnight with CD19CAR CTLs at a 1:1 E:T ratio. The supernatants were collected and the amount of IFN-γ released was measured using ELISA. The CD19^−^ NHL line, BCBL-1, M202 melanoma line, CD19CAR CTLs, and PBLs were used as controls. Samples were set up in triplicate. The results are presented as means ± SEMs of two independent experiments.

To understand the potential underlying mechanism of resistance, we first performed a recognition assay. CD19CAR CTL-sensitive parental cells were efficiently recognized by CD19CAR CTLs as measured by IFN-γ release from CTLs. Despite their differential sensitivity to CD19CAR CTL killing (Figure 2), CD19CAR CTL-resistant Ramos R, Raji R, and Daudi R NHL sublines were also recognized by CD19CAR CTLs (Figure 3A). The amount of IFN-γ release from CD19CAR transduced CLLs upon co-culture with resistant NHL cells was comparable to those upon recognition of parental cells. A fluorescence-activated cell sorting (FACS) analysis revealed that CD19CAR CTL-resistant cells express comparable levels of surface CD19/CD20 expression to those of their parental counterparts (Figure 3B). These results indicate that the recognition machinery (CD19 receptor) of NHL-R cells was intact, but these cells had developed cross-resistance to apoptotic death signals delivered by CD19CAR CTLs and other effector cells, possibly via alterations in the dynamics of intracellular signaling networks.

### 2.4. HDACi (SAHA and LBH589) and Celecoxib (Celebrex) Reverse Resistance to CD19CAR CTL Killing

**Figure 4:**
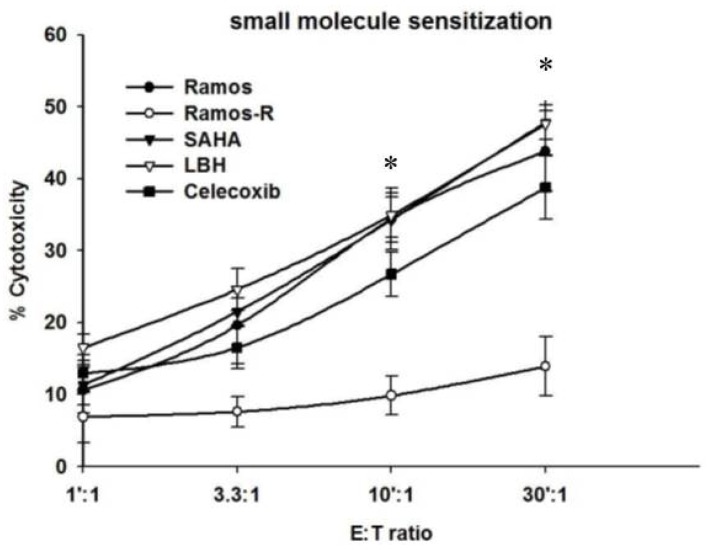
HDACi (SAHA and LBH589) and celecoxib (Celebrex) reverse NHL resistance to CD19CAR CTL killing. CD19CAR CTL-resistant Ramos cells (10^6^) were left either untreated or pretreated with suberoylanilide hydroxamic acid (SAHA) (1.0 μmol/L), panobinostat (LBH) (0.5 μmol/L), and celecoxib (5.0 μmol/L) for 48 h. The cells were then washed 2× and labeled with ^51^Chromium (1 h, 37 °C). Thereafter, cells were washed 2× with ice cold PBS and used in a standard ^51^Cr-release assay using CD19CAR CTLs as effectors. The results are presented as means ± SEMs of duplicate samples. * *p* values < 0.05 were considered to be significant.

HDACi and celecoxib regulate the expression patterns of apoptotic genes, rendering tumors that are more susceptible to apoptotic stimuli [31,32,33,35,42,43,45] and can overcome immune-resistance. We tested whether these small molecules could reverse the resistance of NHL cells to CD19CAR CTL killing. The incubation of CD19CAR CTL-resistant Ramos R sublines with SAHA (1.0 μmol/L), LBH589 (0.5 μmol/L), and celecoxib (5.0 μmol/L) for 48 h largely reversed their resistance to CD19CAR CTL (Figure 4). These results suggest that the chromatin remodeling drugs, SAHA and LBH589, and the anti-inflammatory drug, Celecoxib, can partially reverse CD19CAR CTL-resistance of Ramos NHL cells. 

### 2.5. HDACi and Celecoxib (Celebrex) Sensitize CD19CAR CTL-Resistant NHLs to TRAIL Killing

The TRAIL antagonistic mAb significantly reduced the rate of NHL killing by CD19CAR CTL (Figure 1C). To explore the contribution of TRAIL in CD19CAR CTL killing, we tested the sensitivity of parental and resistant NHLs to rhTRAIL. The sensitivity of CD19CAR CTL-resistant NHL cells to TRAIL-mediated killing was significantly reduced compared to the parental line. Pretreatment of CD19CAR CTL-resistant Ramos cells with SAHA (1.0 μmol/L) and celecoxib (5.0 μmol/L) for 48 h partially restored TRAIL sensitivity (Table 1). These results show that CD19CAR CTL-resistant NHLs develop cross-resistance to TRAIL-mediated killing, which suggests TRAIL may be the principal apoptotic pathway employed by CD19CAR CTLs. The short-term pretreatment of NHLs with SAHA and celecoxib partially reversed the resistance.

## 3. Discussion

CD19CAR therapy has shown potential as an alternative cancer therapy. In a phase I/II trial, forty-one NHL patients were administered CD19CAR T cells containing a defined ratio of CD8^+^ and CD4^+^ CAR T cells; an objective response rate (ORR) of 84% and a complete response rate (CR) of 47% were observed in those who received CD19CAR T cells in addition to cyclophosphamide and fludarabine conditioning regimen [46]. A phase I trial of chemo-refractory NHL patients who underwent CAR T cell therapy following autologous hematopoietic stem cell transplantation (HSCT), showed modest results [47]. In another NHL trial, out of eight patients who received HSCT and CD19CAR T cells, three (38%) had a CR and two (25%) had a partial response (PR); in a second trial, out of eight patients, six (75%) had a CR and two (25%) had a PR [47]. 

The first objective of the present study was to understand the dynamics of CD19CAR CTL killing of human NHL lines. CD19CAR transduced primary human CTLs efficiently killed the CD19^+^ NHL lines, Ramos, Raji, Daudi, but not the CD19^−^ line, BCBL-1. CD19-specific killing of transduced cells was further confirmed by blockade of CD19 using antagonistic mAbs. We also noticed that pan-caspase inhibitor (zVAD-fmk) treatment of NHLs significantly reduced the rate of killing, suggesting that CD19CAR CTLs mainly kill CD19-expressing sensitive NHLs through the induction of apoptosis (Figure 1). 

We further expanded the study to other immune effector cells. CD19^+^ NHLs were also efficiently killed by CD19CAR transduced Jurkat human T cells (sorted to 100% purity). While non-activated Jurkat cells were incapable of killing NHLs, hyper-activation of Jurkat cells by high-dose (3000 IU/mL) IL-2 or LAK cells (3000 IU/mL IL-2; 6–8 days) efficiently killed CD19^+^ NHLs. Activated Jurkat and LAK cells kill in a non-discriminate, non-MHC, Ag-independent manner, suggesting that hyper-activation of naïve immune effector cells can kill NHLs regardless of antigen-specificity. However, the utilization of these effector cells is hampered by their potential off-target activities as well as the severe side effects of high-dose IL-2. The specificity of our model was further shown using the CD19^−^ BCBL-1 line, which was not killed by CD19CAR CTLs or the CD19CAR Jurkat line. However, hyper-activated CD19CAR Jurkat (IL-2: 3000 IU/mL) bypassed the antigen-specificity (e.g., CD19) requirement of transduced cells and killed BCBL-1 cells; this was comparable to hyper-activated Jurkat cells. Apparently, hyperactivated CD19CAR transduced or non-transduced Jurkat cells act in a non-discriminate manner similar to LAK cells.

Immune effector cells eradicate tumors mainly through the induction of apoptosis, mediated by TRAIL, FasL, Granzyme, or TNF-α pathways. The main operative apoptotic pathway in CD19CAR transduced CTLs is unknown. To define the principle apoptotic pathway used by CD19CAR CTLs, we treated NHLs with TRAIL antagonistic mAb prior to incubation with effector cells. A significant reduction in killing was observed, suggesting that CD19CAR CTLs primarily employ the TRAIL apoptotic pathway to kill sensitive NHLs. This notion was further reinforced by the observation that R-NHL cells developed cross-resistance to rhTRAIL. The possibility of involvement of other apoptotic pathways is not ruled out, which warrants further studies. Nevertheless, this observation is very important as TRAIL or agonistic TRAIL death receptor (DR4, DR5) mAbs are being clinically used and may be incorporated as adjuvants in CD19CAR CTL-based treatment protocols. 

The modest to low response rates observed in clinical trials might be due to the acquisition of resistance mechanisms by NHLs following the initial infusion of transduced CTLs to avoid CD19CAR CTL-induced apoptosis. The potential underlying mechanism(s) of, and approaches to overcoming, acquired anti-CD19CAR T cell-resistance in NHL remain elusive. Down-regulation or shedding of tumor associated antigens is a potential mechanism of resistance. In fact, CD19 antigen expression on tumor cells might become lost or down-regulated after CD19 CAR T cell infusion [48]. In a separate study, three patients who had ALL relapsed after the early loss of CD19CAR T-cells at 2 weeks to 3 months, and the relapsed ALL cells remained CD19-positive [21]. 

The second objective of this study was to establish an in vitro model of NHL resistance to CD19CAR T cells to mimic in vivo situations. To decipher the dynamics resistance acquisition, we analyzed several anti-CD19CAR CTL-resistant NHL sublines (R-NHL) derived from the serial exposure of sensitive parental lines to excess numbers of anti-CD19CAR CTLs, followed by a limiting dilution analysis to obtain a homogenous population. Dual immunostaining revealed that R-NHL cells retained the surface expression of CD19 and CD20 B cell markers at levels comparable to parental cells. We further performed a recognition assay which showed comparable levels of type I cytokine (IFN-γ) secretion by CD19CAR CTLs upon recognition of sensitive and R-NHL sublines. Thus, the recognition unit on R-NHLs (CD19 surface marker) is not down-regulated/lost during the acquisition of resistance. However, despite efficient recognition by CD19CAR CTLs, R-NHLs became resistant to CD19CAR transduced human primary CTLs. R-NHLs further developed cross-resistance to CD19CAR Jurkat (sorted to 100% purity), activated Jurkat, and LAK cells. These results suggest that the development of resistance is autonomous of loss or down-regulation of CD19 and might be due to aberrant apoptotic machinery. In fact, preliminary experiments (which require further scrutiny) showed the aberrant expression of several apoptotic genes in R-NHLs (data not shown). The development of cross-resistance implies that the prolonged exposure of NHLs to CD19CAR CTLs results in the selective outgrowth of NHLs which lose the ability to undergo apoptosis in response to cytotoxic stimuli delivered by various immune effector cells. The development of cross-resistance further suggests the use of common apoptotic machinery by immune effectors to kill NHLs. Thus, designing approaches to modulate deregulated apoptotic machinery could potentially override resistance. 

Our last objective was to design novel approaches to overcome acquired resistance. Abnormal levels of pro- and anti-apoptotic proteins expressed in tumors might be responsible for apoptosis resistance. Whether the extrinsic and intrinsic apoptotic pathways are initiated or halted depends on the balance between the expression levels of pro- and anti-apoptotic proteins. A common trend found among various types of cancers is a decrease in pro-apoptotic Bax and Bak and an increase in anti-apoptotic Bcl-2, Mcl-1, and Bfl-1 levels [49,50]. Bcl-2 inhibitors restore normal extrinsic and intrinsic apoptosis pathways in resistant tumors. For instance, ABT-737, a small molecule BH3 mimetic, effectively kills ALL blast cells by disrupting the Bcl-2/Bax complex [51]. Navitoclax, another BH3 mimetic, has great activity against CLL [52]. Preliminary data suggest that R-NHLs have distorted expression levels of apoptotic proteins [53]. Thus, the restoration of the expression of apoptotic regulators towards a proapoptotic milieu can sensitize R-NHLs to CD19 CAR CTLs. Several strategies have been designed to override the resistance mechanisms of NHLs, including the use of drugs with known anticancer properties, such as histone deacetylase inhibitors (HDACis) and celecoxib. These drugs regulate the expression patterns of apoptotic genes rendering tumors more susceptible to apoptotic stimuli [31,32,33,35,42,43] and overcoming immune resistance. Thus, we tested whether celecoxib and HDACi can sensitize R-NHLs to CD19 CAR CTLs. Our results indicate that the short-term exposure of R-NHLs to clinically achievable and non-toxic concentrations of SAHA, LBH589, and celcoxib largely reversed their resistance to CD19CAR CTL (Figure 4). These results suggest that chromatin remodeling drugs (SAHA, LBH589) and the anti-inflammatory drug, celecoxib, can partially reverse the CD19CAR CTL-resistance of R-NHLs. Moreover, SAHA and celecoxib sensitized R-NHL cells to rhTRAIL. These FDA-approved drugs can be safely used in clinical settings of NHL therapy. 

NHLs could potentially undergo alternative differentiation and signaling pathways to avoid recognition by CD19CAR T cells. For instance, the biopsy of a patient with plasmablastic lymphoma (PBL) had tumor cells lacking CD19 and other markers of pre-plasmacytic B-cell differentiation, suggesting that PBL might have used alternative B-cell differentiation pathways to proliferate and avoid apoptosis [54]. Also, mutations in genes that code for key regulators of cell proliferation and survival pathways, such as BRAF, NRAS, Apaf-1, and p53 genes, might also play roles in resistance [55,56,57]. Aberrant BCR signaling pathways, including those involving the Src family kinases (Lyn, Syk), Akt/mTOR, Btk, NF-κB, observed in CLL and NHL [58,59] might also be implicated in NHL clinical resistance. These possibilities require detailed investigation. 

The identity of the main apoptotic regulator(s) whose distorted expression level(s) renders NHLs resistant to CD19 CAR CTLs remains to be elucidated. Moreover, the underlying molecular mechanism(s) by which HDACi and Celebrex modulate the expressions of these proteins towards pro-apoptotic profiles and sensitize R-NHLs has yet to be uncovered. Though further investigation, including the establishment of an animal model, is required, our in vitro data provides a strong platform and rational molecular/biological basis for the incorporation of FDA-approved drugs with minimal toxicity into clinical protocols of CD19CAR CTL-based therapy of NHL. 

## 4. Materials and Methods

### 4.1. Cell Lines and Sublines

Human non-Hodgkin’s Lymphoma Ramos, Raji, and Daudi cell lines were obtained from Dr. Martinez-Maza (UCLA, Jonsson Comprehensive Cancer Center). For the generation of CD19CAR CTL R-NHL lines, parental (P) cells were grown in the presence of increasing (step-wise) numbers of CD19CAR CTLs for a total of 8 weeks (1–2 weeks for each E:T). Forty percent to 60% of the NHL cells survived the first cycle of selection (10:1, 2 weeks), and the percentage of dead cells drastically reduced during subsequent selection cycles until no further killing was observed. Remaining viable NHL cells were then subjected to two consecutive rounds of limiting dilution analysis. Single cells were propagated and maintained in RPMI-1640 supplemented with 10% (*v*/*v*) heat-inactivated fetal bovine serum (FBS). After immune selection, sublines were maintained in a medium containing excess (10:1) CD19CAR CTLs and were grown in CD19CAR CTL-free medium for at least 1 week prior to analysis. Cultures were incubated in a controlled atmosphere incubator at 37 °C with a saturated humidity at 0.5 × 10^6^ cells/mL and were used once they had reached logarithmic phase for each experiment. Cultures were routinely checked for mycoplasma contamination (Lonza, Basel, Switzerland).

### 4.2. Reagents

Blocking Abs and fluorochrome (FITC and PE) conjugated monoclonal antibodies (mAb) specific to CD19 or CD20 receptors were purchased from eBiosciences (San Diego, CA, USA) to be used in the FACS analyses. Soluble recombinant human TRAIL (rhTRAIL) was purchased from Peprotech (Rocky Mountains, NJ, USA). LBH589, SAHA, and celecoxib was procured commercially. Stock solutions were diluted in dimethyl sulfoxide (DMSO) at a final concentration of 10 mM (kept at −80 °C). Working solutions were diluted in 1× sterile PBS and used in assays. The final concentration of DMSO did not exceed 0.1% in any experiment. 

### 4.3. Transduction of CD8 CTLs with CD19CAR Retrovirus

A non-adherent population of healthy donor human peripheral blood mononuclear cells (PBMC) was cultured in AIM-V media supplemented with 5% human AB serum, αCD3 antibody (OKT3; 50 ng/mL), and IL-2 (300 IU/mL) for 48 h. CD3^+^CD8^+^ CTLs were isolated by EasyStep Negative Selection enrichment kits (Stem Cell Technologies, Vancouver, BC, Canada) as per the manufacturer’s instructions. CTLs were transduced with the CD19CAR vector. CD8^+^ CTLs with more than 95% CD19CAR expression were used in all experiments.

### 4.4. Cell-Mediated Cytotoxicity Assay

NHL cells were washed once with cold 1× PBS and labeled with 100 μCi of Na_2_^51^CrO_4_ for 1 h (37 °C/5%CO_2_). After 3× washes, 10^4^ cells were added to V-bottom 96-well plates and used immediately, as previously described. The percentage of specific ^51^Cr-release was measured as % cytotoxicity = (experimental release − spontaneous release)/(total release − spontaneous release) × 100.

### 4.5. Surface CD19/CD20 Expression

NHL cells (2 × 10^6^) were washed 2× with ice-cold 1× PBS and stained with 0.5 μg mouse anti-human CD19 or CD20 mAb (eBiosciences, San Diego, CA, USA) or an isotype control (pure IgG1; 20 min on ice, light protected). Then, the cells were washed 2× with ice-cold 1× PBS, stained with FITC-labeled secondary antibody (30 min on ice, light protected) and subjected to fluorescence-activated cell sorting (FACS) analysis.

### 4.6. Measurement of Transduction Efficiency of CD19CAR Transduced CTLs

Transduced and non-transduced T cells were harvested and washed with FACS buffer (PBS + 5% FCS and 10% sodium azide). An aliquot was used to assay for cell viability with the trypan blue dye exclusion assay. Staining of the CD19 CAR construct was performed using a 1:5 dilution of Affinpure goat-anti mouse IgG (Jackson ImmunoResearch, West Grove, PA, USA, Cat# 115-065-072) diluted in FACS buffer. Cells were incubated for 30 min at 4 °C in this mixture, washed 2× with FACS buffer, and blocked for 20 min at 4 °C using a 1:10 dilution of mouse gamma globulin (Jackson ImmunoResearch, Cat# 015-000-002). Excess blocking was washed with FACS buffer, and cells were stained with PE streptavidin (BD Biosciences, San Jose, CA, USA, Cat# 349023) (20 min, 4 °C). Cells were washed 2× with FACS buffer and resuspended in the same buffer containing propidium iodide. Cells were subsequently analyzed by flow cytometry using an LSRII flow cytometer, BD (Franklin Lakes, NJ, USA).

### 4.7. Statistical Analysis

Assays were set up in duplicate or triplicate, and results are expressed as means ± standard error of the means (SEM). The statistical analyses involved two-tailed paired *t* tests with confidence intervals (CI) of 95% to determine the significance of differences between treatment groups (*p* < 0.05: significant). ANOVAs were used to test the significance among the groups using InStat 2.01 software (https://www.graphpad.com/scientific-software/instat/).

## 5. Conclusions

CD19CAR-redirected CTL immunotherapy is a novel approach in clinical oncology which has shown initial promising results in the clinical trials of B cell malignancies. However, there are certain drawbacks that require further examination. One such limitation is the low clinical response rate observed in patients which might be due to the adoption of various resistance mechanisms by tumor cells. Our results suggest that during the acquisition of resistance to CD19CAR-redirected CTL therapy, NHLs maintain CD19 surface expression and can be efficiently recognized by CD19CAR CTLs. However, despite their efficient recognition, highly efficient CD19CAR CTLs fail to kill NHLs most probably due to distorted apoptotic machinery in NHLs. Subtoxic concentrations of FDA-approved drugs with broad gene regulatory effects, such as HDACi and Celebrex, modulate the expression profiles of apoptotic genes and restore the sensitivity of NHL lines to CD19CAR CTLs. Our in vitro results, while requiring further investigation, provide strong rationale for the incorporation of small molecule sensitizers into the clinical protocols of CD19CAR CTL immunotherapy of NHL patients. 

## Figures and Tables

**Table 1:** Sensitization of CD19CAR CTL-resistant Ramos cells to rhTRAIL-induced killing by SAHA and celecoxib.

Cell Line	TRAIL (ng/mL)			
	0	10	25	50
Ramos Parental	196.7 ± 2.6 (4.6%)	125.3 ± 7.1 (37.35%)	81.7 ± 3.8 (59.15%)	68.3 ± 3.5 (65.85%)
Ramos resistant (R)	192.7 ± 3.2 (3.65%)	185.3 ± 4.8 (7.35%)	179.7 ± 4.4 (10.15%)	171.3 ± 3.4 (14.35%)
Ramos R +Celecoxib	187 ± 3.8 (6.5%)	140.1 ± 2.1 (29.95)	120.3 ± 1.8 (39.85%)	108 ± 2.6 (46%)
Ramos R + SAHA	190.7 ± 2.7 (4.65%)	137.3 ± 4.4 (31.35%)	119 ± 2.9 (40.5%)	97.7 ± 3.8 (51.15%)

NHL cells were left either untreated or pretreated with SAHA (1.0 μmol/L) or celecoxib (5.0 μmol/L) (48 h). Cells were then washed 2× and incubated with rhTRAIL (0–50 ng/mL, 18 h). Trypan blue dye exclusion assay was used to measure the number of viable cells. Results are presented as means ± SEMs of three independent experiments. Values in parentheses indicate the percentages of dead cells (200 cells were counted for each condition). In all three rhTRAIL concentrations used (10, 25, 50 ng/mL) there was statistical significance between the rate of killing of drug-treated (Celebrex or SAHA) Ramos R cells compared to Ramos R cells in the absence of any drugs (control). In addition, there was statistical significance (*p*-values < 0.05 were considered to be significant) between Ramos parental and Ramos R cells treated with TRAIL. Moreover, there was statistical significance between combinations of Ramos + SAHA or Ramos + Celebrex compared to TRAIL + SAHA or TRAIL + Celebrex. *p*-values < 0.05 were considered to be significant.
